# Prehospital management of burns requiring specialized burn centre evaluation: a single physician-based emergency medical service experience

**DOI:** 10.1186/s13049-020-00771-4

**Published:** 2020-08-20

**Authors:** Ludovic Maudet, Mathieu Pasquier, Olivier Pantet, Roland Albrecht, Pierre-Nicolas Carron

**Affiliations:** 1 Faculty of Biology and Medicine, University of Lausanne, Rue du Bugnon 21, CH-1011 Lausanne, Switzerland; 2 Department of Emergency Medicine, Lausanne University Hospital, Rue du Bugnon 46, CH-1011 Lausanne, Switzerland; 3 Department of Anesthesiology, Lausanne University Hospital, Ru du Bugnon 46, CH-1011 Lausanne, Switzerland; 4 Department of Intensive Care Medicine and Burn Centre, Lausanne University Hospital, Rue du Bugnon 46, CH-1011 Lausanne, Switzerland; 5 Rega – Swiss Air-Rescue, Rega Centre, PO Box 1414, CH-8058 Zurich, Switzerland

**Keywords:** Burn injury, Burn size, Emergency medical services, Fluid therapy, Pain management, Prehospital

## Abstract

**Background:**

Emergency medical services regularly encounter severe burns. As standards of care are relatively well-established regarding their hospital management, prehospital care is comparatively poorly defined. The aim of this study was to describe burned patients taken care of by our physician-staffed emergency medical service (PEMS).

**Methods:**

All patients directly transported by our PEMS to our burn centre between January 2008 and December 2017 were retrospectively enrolled. We specifically addressed three “burn-related” variables: prehospital and hospital burn size estimations, type and volume of infusion and pain assessment and management. We divided patients into two groups for comparison: TBSA < 20% and ≥ 20%. We a priori defined clinically acceptable limits of agreement in the small and large burn group to be ±5% and ± 10%, respectively.

**Results:**

We included 86 patients whose median age was 26 years (IQR 12–51). The median prehospital TBSA was 10% (IQR 6–25). The difference between the prehospital and hospital TBSA estimations was outside the limits of agreement at 6.2%. The limits of agreement found in the small and large burn groups were − 5.3, 4.4 and − 10.1, 11, respectively. Crystalloid infusion was reported at a median volume of 0.8 ml/kg/TBSA (IQR 0.3–1.4) during the prehospital phase, which extrapolated over the first 8 h would equal to a median volume of 10.5 ml/kg/TBSA. The median verbal numeric rating scale on scene was 6 (IQR 3–8) and 3 (IQR 2–5) at the hospital (*p* < 0.001). Systemic analgesia was provided to 61 (71%) patients, predominantly with fentanyl (*n* = 59; 69%), followed by ketamine (*n* = 7; 8.1%). The median doses of fentanyl and ketamine were 1.7 mcg/kg (IQR 1–2.6) and 2.1 mg/kg (IQR 0.3–3.2), respectively.

**Conclusions:**

We found good agreement in burn size estimations. The quantity of crystalloid infused was higher than the recommended amount, suggesting a potential risk for fluid overload. Most patients benefited from a correct systemic analgesia. These results emphasized the need for dedicated guidelines and decision support aids for the prehospital management of burned patients.

## Background

The incidence of burn injuries is lowering in high-income countries [1]. However, despite prevention efforts, the social and economic costs remain high and individual consequences are serious [2, 3]. Severe burns require specific expertise and significant resources throughout the course of care [4]. Evidence-based medicine literature supporting prehospital recommendations or guidelines is scarce, although critical issues are shared by many, if not all, emergency services [5]. Keeping the victim stable and orienting care towards a specialised unit are the main priorities of prehospital care of burned patients [6], as well as assessing any possible concomitant injury (inhalation injuries, carbon monoxide or cyanide toxicity and trauma) [7, 8]. Oxygenation and sometimes intubation in patients with suspected smoke inhalation or impaired consciousness [9, 10], co-intoxication treatment, analgesia, and protection against hypothermia are indicated on site [11, 12]. On the other hand, prehospital recommendations are inconsistent regarding the method and accuracy of the estimation of the total burned surface area [13–17], the amount of fluid volume to be infused precociously [18] and the type of analgesia to be administered [19].

In this study, we wanted to analyse the prehospital management of burned patients in a Swiss physician-staffed emergency medical service (PEMS), focusing on the three following key points: the burned surface area estimation, prehospital fluid administration, and analgesia management.

## Methods

### Study setting and design

The PEMS of the Lausanne University Hospital includes one ground-based emergency resuscitation vehicle and a rescue helicopter from the Swiss Air-Ambulance. These two PEMS teams operate respectively on ground in the Lausanne city (150 km^2^) and the surrounding area (400 km^2^) for about 400,000 inhabitants, and by air (rescue helicopter) in a mixed urban and countryside area of 5700 km^2^ for about 1,300,000 inhabitants. The prehospital emergency physicians are all trained in emergency medicine, including experiences in anaesthesiology and intensive care. All benefited from specific theoretical training in the prehospital management of burns. Trained paramedics constituted the initial response on site. Prehospital emergency physicians were dispatched primarily according to specific keywords (in the case of life-threating emergencies) from a single emergency dispatch centre [20] or upon request by the paramedics on site. In our setting, the two analgesic drugs available were fentanyl (Fentanyl-Janssen®, 50 mcg/ml, Janssen-Cilag AG, Zoug, Switzerland) and racemic ketamine (Ketamin Sintetica®, 50 mg/ml, Sintetica SA, Mendrisio, Switzerland).

This cohort study was based on the retrospective review of prospectively collected standardized data of the Lausanne PEMS. We screened all missions from January 2008 to December 2017. During this period, the prehospital care of burned casualties by the Lausanne PEMS were homogeneous and guided by specific local recommendations. We included all patients suffering from thermic burn, directly transported by the Lausanne PEMS, and treated at the Lausanne University Hospital Burn Centre, regardless of their age. The Lausanne University Hospital is a tertiary referral hospital receiving 65,000 emergency patients annually, serving a population of 1,500,000 and is considered a Level 1 trauma centre. It is also one of the two burn centers in Switzerland (the other being at Zurich University Hospital) accredited by the European Burn Association. In accordance with federal regulations, severe burns are systematically referred to these specialized centers by every emergency medical service, without direct contribution or validation from burn specialists during the prehospital management. In 2017, the Lausanne University Hospital Burn Centre took care of 82 severe burned adults and children, with an average length of stay in the burn specialized intensive care unit (6 beds) of 0.2 day in children and 0.8 day in adults per percentage of TBSA. We excluded chemical or electrical burns and secondary missions (inter-hospital transfers). The Burn Centre database was also consulted to identify patients managed by the Lausanne PEMS and who may have been missed by our initial search strategy and for complementary missing data.

### Data collection

The data collection was approved by the institutional ethical committee (CER-VD N° 2016–01433). Data were extracted from the prehospital and hospital electronic records. Patients were aggregated in two groups based on a 20% TBSA cut-off, which distinguishes large burns (≥ 20%) from smaller ones (< 20%). This cut-off is a recognized threshold for beginning an aggressive reanimation and orienting patients in specialized centres [4, 21]. The separation into two subgroups was based on the prehospital TBSA estimation, except in the case of a missing value (5), where the hospital TBSA estimation was considered.

The demographic characteristics collected were age (in years), gender and estimated in-hospital weight (in kilograms). Among the background of injury, domestic burns occurring at home were distinguished from burns occurring during work or leisure activities. The following burn-related acute comorbidities were also collected: facial burn, inhalation injury and carbon monoxide intoxication.

The type of PEMS (ground-bases vs. helicopter) was collected, as well as the following time intervals: response interval (from dispatch centre alarm to PEMS arrival on scene), on-scene interval (from PEMS arrival to departure) and transport interval (departure from the scene to arrival at hospital) [22]. We defined the prehospital treatment interval as the time interval from PEMS arrival on-scene to hospital arrival. The severity of involvement was coded and reported by the prehospital emergency physician according to the prehospital National Advisory Committee for Aeronautics (NACA) score [23].

The initial following parameters were recorded on-scene and at the hospital: respiratory rate, heart rate, blood pressure, blood oxygen saturation, Glasgow coma score (GCS), and capillary refill time (CRT, defined as abnormal if > 2 s).

Our main outcome was to analyse and describe the following three “burn-related” variables: burned surface area estimation, volume of liquid infused during the prehospital treatment interval, and the prehospital analgesia provided.

We also reported the following prehospital interventions: oxygen therapy, tracheal intubation and vascular access.

The prehospital TBSA estimation (based on the rule of nines) reported by the PEMS was compared to the hospital TBSA estimation (based on Lund and Browder chart) reported by the specialist surgeon at the Burn Centre after the first wound treatment session. Only burned surfaces involving second or higher degree were considered for both prehospital and in-hospital TBSA estimation. We used the volume of prehospital infusion together with the patient’s weight to calculate the volume infused per kilogram of body weight and TBSA (ml/kg/TBSA). We further used this data with a hypothetical duration of 8 h to compare the extrapolated volume infused in our prehospital setting to the Parkland formula [24]. Patients of all TBSA estimates were included in this analysis, even though the Parkland formula is generally recommended for burns involving ≥20% TBSA. The pain was assessed and reported using the verbal numeric rating scale (VNRS, ranging from 0 to 10). The doses and route (intravenous, intramuscular or intranasal) of prehospital analgesia were also analysed, and evaluated by body weight (mcg/kg for fentanyl and mg/kg for ketamine) and compared to the dosages usually recommended for acute analgesia in trauma or burn patients.

Outcome measures included the mortality, considered as the occurrence of death during the acute care period, from prehospital physician arriving on-scene to patient leaving hospital after initial care. We also recorded the severity of injuries according to the Injury Severity Score (ISS), length (days) of hospital stay and length (days) of intensive care unit (ICU) stay.

### Statistical analysis

We used Stata (Stata/IC 14.2, StataCorp LLC, TX77845, USA) software to perform the statistical analysis. Descriptive statistics included numbers and frequencies for categorical variables and medians and interquartile ranges (25th to 75th percentile) for continuous variables. The chi-square test was used for categorical data comparisons, while non-normally distributed continuous data were tested using the Wilcoxon test. A bilateral *p*-value < 0.05 was considered to indicate a significant difference. A Bland-Altman plot was used to compare the prehospital and hospital TBSA estimations [25]. We a priori defined clinically acceptable limits of agreement in the small and large burn groups to be ±5% and ± 10%, respectively.

## Results

### Characteristics of the study population

**Fig. 1:**
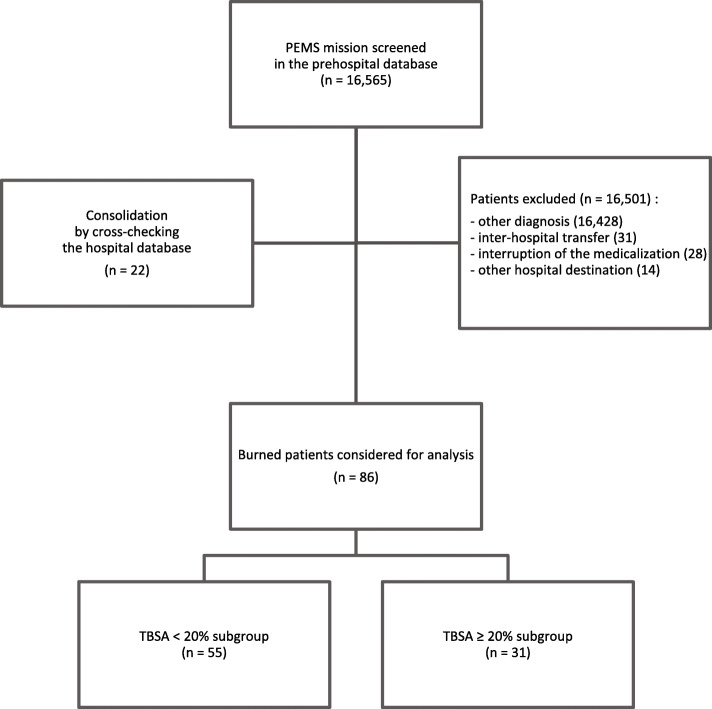
Flow chart of the case load selection (1 January 2008 to 31 December 2017). The separation into two subgroups is based on the prehospital TBSA estimation, except in the case of a missing value (5), where the hospital TBSA estimation is considered

Among the 16,565 PEMS missions screened during the 10-year study period (Fig. 1), we included 86 patients. Patients’ characteristics according to the burn severity subgroups are reported in Table 1**.** While the burn injury context (mostly domestic accidents) did do not differ between groups, patients suffering large burns were older (23 vs. 39 years old; *p* = 0.031), more prone to suffer from inhalational injury or carbon monoxide intoxication (27% vs. 65%; *p* = 0.001) and had longer PEMS on-scene times (16 vs. 28 min; *p* < 0.001). No patient in the study group suffered from major trauma or multiple traumatic injuries (bone or abdomino-thoracic injuries).

**Table 1:** Study population and missions characteristics. Data are expressed as numbers and frequencies for categorical variables and medians and interquartile ranges (25th to 75th percentile). [] = missing values

	Study group *n* = 86 (100%)	TBSA < 20% *n* = 55 (64%)	TBSA ≥20% *n* = 31 (36%)	*p*-value
Age (years) [0]	26 (12–51)	23 (3–50)	39 (20–58)	0.031*
Gender male, n (%) [0]	63 (73)	43 (78)	20 (65)	0.169
Weight (kg), median [11]	65 (20–81)	62 (14–88)	67 (60–75)	0.253
Injury context, n (%) [0]:				
- Domestic	50 (58)	33 (60)	17 (55)	0.641
- Work	18 (21)	11 (20)	7 (23)	0.778
- Leisure	6 (7)	5 (9.1)	1 (3)	0.305
- Others	11 (13)	5 (9.1)	6 (19)	0.171
Facial burn, n (%) [0]	52 (60)	29 (53)	23 (74)	0.051
Inhalation injury, n (%) [0]	35 (41)	15 (27)	20 (65)	0.001*
Carbon monoxide intoxication, n (%) [0]	4 (4.7)	0	4 (13)	0.006*
Type of PEMS, n (%) [0]:				
- Ground	35 (41)	23 (42)	12 (39)	0.381
- Helicopter	47 (55)	32 (58)	15 (48)	0.778
- Both	4 (4.7)	0	4 (13)	0.006*
Time intervals (minutes), median [0]:				
- Response	15 (9–21)	15 (10–21)	13 (9–20)	0.342
- On-scene	19 (13–30)	16 (11–22)	28 (17–42)	< 0.001*
- Transport	11 (7–17)	11 (6–18)	10 (7–16)	0.790
- Prehospital treatment	33 (22–45)	29 (19–39)	45 (24–56)	0.003*
NACA score, median [0]	4 (3–5)	4 (3–4)	5 (4–5)	< 0.001*

The on-scene heart and respiratory rates were higher and the blood oxygen saturation was lower in patients suffering large burns, while this was not the case at hospital admission (Table 2). Overall intubation rate was 24% (6 (11%) patients in the small and 15 (48%) patients in the large burn subgroups) (*p* < 0.001).

**Table 2:** Vitals signs and non-specific interventions. Data are expressed as numbers and frequencies for categorical variables and medians and interquartile ranges (25th to 75th percentile). [] = missing values

	Study group *n* = 86 (100%)	TBSA < 20% *n* = 55 (64%)	TBSA ≥20% *n* = 31 (36%)	*p*-value
Vital parameters on scene:				
- Respiratory rate (min^− 1^) [4]	20 (16–25)	18 (16–22)	25 (20–30)	< 0.001*
- SpO_2 (_%) [8]	98 (95–100)	98 (96–100)	97 (92–99)	0.01*
- Pulse rate (min^− 1^) [7]	100 (85–116)	90 (80–105)	106 (100–120)	< 0.001*
- Systolic blood pressure (mmHg), [13]	140 (90–150)	140 (90–150)	140 (90–150)	0.735
- Capillary refill time ≥ 2 s, n (%) [0]	6 (7)	4 (7.3)	2 (6.5)	0.886
- GCS [0]	15 (15–15)	15 (15–15)	15 (15–15)	0.065
Vital parameters at ED arrival:				
- Respiratory rate (min^− 1^) [29]	16 (12–20)	16 (13–20)	13 (12–21)	0.106
- SpO_2_ (%) [22]	99 (96–100)	99 (96–100)	99 (96–100)	0.949
- Pulse rate(min^− 1^) [18]	100 (80–110)	92 (78–105)	103 (90–111)	0.061
- Systolic blood pressure (mmHg) [24]	89 (66–98)	84 (66–96)	90 (62–100)	0.821
- GCS [18]	15 (3–15)	15 (15–15)	3 (3–15)	< 0.001*
Oxygen therapy, n (%) [8]	51 (65)	25 (51)	26 (90)	0.001*
Tracheal intubation, n (%) [0]	21 (24)	6 (11)	15 (48)	< 0.001*
Vascular or intraosseous access, n (%) [1]	64 (75)	38 (69)	26 (87)	0.073

Vascular or intraosseous access were done similarly in both subgroups, with an overall occurrence of 64 cases (75%; *p* = 0.073).

### Burn-specific assessments and interventions

**Fig. 2:**
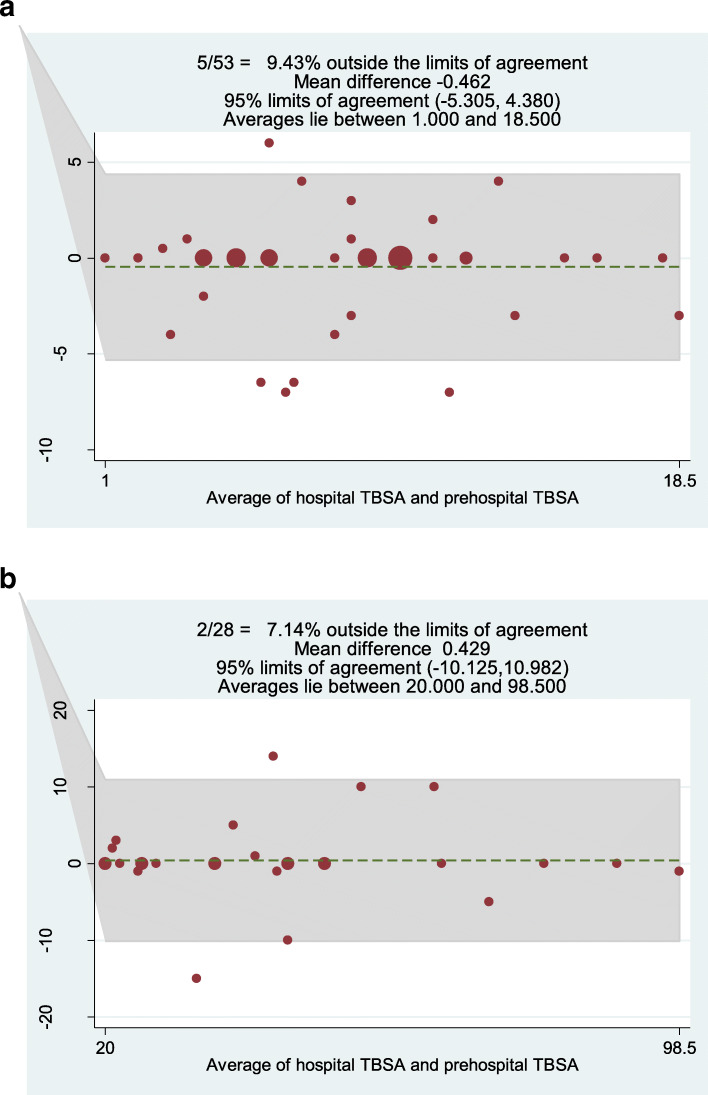
Bland-Altman analysis of the differences between the hospital and prehospital TBSA estimations in the small (Fig. 2**a**) and large (Fig. 2**b**) burns groups. For each comparison, the mean value between the two estimations is plotted against their difference. The mean difference between the hospital and prehospital TBSA estimations were − 0.462 for the small and 0.429 for the large burns groups. The lower and upper limits of agreement were − 5 and 4% in the small burns group and − 10 and 11% in the large burns group

Prehospital estimation of the TBSA was reported by the PEMS in 81 of the 86 cases (94%). The median TBSA was 10% (IQR 6–25). Fifty-five patients (64%) suffered from small burns (TBSA < 20%) and 31 (36%) patients suffered from large burns (TBSA ≥20%). On average, prehospital TBSA estimation was slightly less than hospital TBSA estimation, but the medians were not statistically different (*p* = 0.78). The mean difference in the small burns subgroup was 0.5%, whereas it was − 0.4 in the large burns subgroup. The limits of agreement between the prehospital and hospital TBSA estimations in both groups are shown in Fig. 2. The limits of agreement between the prehospital and hospital TBSA estimations for all patients are available as a supplementary file.

Sixty-four (75%) patients received intravascular infusion during the prehospital phase (median duration of 33 min; IQR 22–45), all with crystalloids and at a median volume of 0.8 ml/kg/TBSA (IQR 0.3–1.4). Extrapolated over the first 8 h, this would equal to a median volume infused of 10.5 ml/kg/TBSA (Table 3).

**Table 3:** Burn-specific assessment and intervention. Data are expressed as numbers and frequencies for categorical variables and medians and interquartile ranges (25th to 75th percentile). [] = missing values

	Study group *n* = 86 (100%)	TBSA < 20% *n* = 55 (64%)	TBSA ≥20% *n* = 31 (36%)	*p*-value
Prehospital (initial) TBSA estimation (in %) [5]	10 (6–25)	9 (5–10)	40 (25–50)	< 0.001*
Hospital (final) TBSA measurement (in %) [1]	10 (6–27)	8.5 (4–10)	43 (25–55)	< 0.001*
Absolute variation of TBSA [5]	0 (0–3)	0 (0–2)	0 (0–4)	0.2093
Crystalloid infusion (ml/kg/TBSA) [33]	0.8 (0.3–1.4)	1.3 (0.8–1.8)	0.3 (0.1–0.4)	< 0.001*
Crystalloid infusion reported to 8 h (ml/kg/TBSA) [34]	10.5 (3.4–17.4)	15.5 (12–25)	2.7 (1.4–4)	< 0.001*
Initial pain score (VNRS) [27]	6 (3–8)	5.5 (3–7)	8 (4–10)	0.039*
Pain score at ED arrival (VNRS) [51]	3 (2–5)	3 (2–5)	4 (3–7)	0.154
Analgesia provision, n (%) [0]	61 (71)	37 (67)	24 (77)	0.32
Intranasal medication, n (%) [0]	9 (10)	8 (15)	1 (3.2)	0.1
Fentanyl administration, n (%) [0]	59 (69)	37 (67)	22 (71)	0.723
Fentanyl dose (mcg/kg) [0]	1.7 (1–2.6)	1.4 (1–1.9)	2.7 (1.7–4.7)	0.002*
Ketamine administration, n (%) [0]	7 (8.1)	1 (1.8)	6 (19)	0.004*
Ketamine dose (mg/kg) [0]	2.1 (0.3–3.2)	2.1	2.4 (0.3–3.2)	1

An initial pain assessment using the VNRS was available for 59 (69%) patients. Pain was significantly higher on site than at hospital arrival (median VNRS of 6 (IQR 3–8) vs. 3 (IQR 2–5), *p* < 0.001). Analgesia was provided in 61 (71%) patients, mostly with fentanyl (59), at a median dose of 1.7 mcg/kg (IQR 1–2.6) (Table 3). Fentanyl was administered intranasally on 9 (10%) occasions, predominantly (*n* = 8, 89%) for patients from the small burns subgroup.

The clinical course and outcomes of patients are presented in Table 4. Seventy-eight out of the 86 patients survived at hospital discharge (91%). Most non-survivors (7/8, 88%) belonged to the large burns group.

**Table 4:** Outcomes. Data are expressed as numbers and frequencies for categorical variables and medians and interquartile ranges (25th to 75th percentile). [] = missing values

	Study group *n* = 86 (100%)	TBSA < 20% *n* = 55 (64%)	TBSA ≥20% *n* = 31 (36%)	*p*-value
ISS [21]	4 (1–21)	1 (1–4)	25 (11–25)	< 0.001*
Death, n (%) [0]	8 (9.3)	1 (1.8)	7 (23)	0.001*
ICU stay, n (%) [1]	53 (62)	24 (44)	29 (94)	< 0.001
ICU length of stay (days) [1]	7 (2–27)	4 (2–6)	22 (7–50)	< 0.001*
Total hospital length of stay (days) [1]	14 (1–28)	5.5 (1–18)	30 (8–88)	< 0.001*

## Discussion

The present study, which included 86 patients, is one of the few to specifically assess the prehospital civilian medical management of burned patients. As in other groups described in Europe, North America and Australia, our patients were predominantly men, of working age, and were injured at home or at work [3, 26, 27]. The agreement between the prehospital and hospital burn size estimations was clinically correct in most cases (within the limits of agreement in 93.8% of cases). The quantity of crystalloid infused in the first hour was higher than the recommended amount, suggesting a potential risk for fluid overload. Most patients benefited from systemic analgesia (with either fentanyl or ketamine) with a significant reduction in pain upon arrival at the hospital.

We devoted specific attention to the comparison of the prehospital and in-hospital TBSA estimations as accurate assessment of the burn size remains a challenge in the prehospital care of burned patients [14–17]. We mostly used the rule of nines in prehospital care, whereas a Lund and Browder chart was used by the burn specialists. Without any well-established cut-off, we set up ±5% and ± 10% to be critical differences between TBSA estimation for small and large burns, respectively, based on a clinical appreciation. The Bland-Altman analysis showed that 9% of the small burns and 7% of the large burns had significant differences between the prehospital and hospital specialized TBSA estimations. The limits of agreement found in the small and large burns groups were − 5.3, 4.4 and − 10.1, 11 respectively, which slightly exceeds the pre-set cut-off. This discrepancy seems to be less than reported by previous studies with similar cut-off (± 5% TBSA) with 40% [28] differences (or even more) between estimations [17, 29]. Small burns TBSA estimations are more prone to errors, as previously shown [30], and small burns tend to be overestimated, whereas the larger ones tend to be slightly underestimated. The maximal error in TBSA estimation represented 82% of the variation from the median TBSA in the small burns subgroup and 35% in the large burns subgroup. This highlights the impact of errors in TBSA estimation regarding the volume to be infused for small burns as determined by the Parkland formula [24].

Precisely regarding the amount of fluid infused, we found that the median quantity of crystalloids was exaggerated. In the small burns subgroup, which (according to the recommendations [19, 21]) does not require aggressive volume expansion, we discovered a seven-fold overhead by correcting the volume infused over 8 h like in the first phase in the Parkland formula. In the large burns subgroup, the infused volume nearly corresponded with the recommendations. This finding is consistent with similar observations made in other studies that described deviation from the Parkland formula from 28 to 61% of the time [14, 28, 30]. This volume overload reported to the time of intervention is potentially the consequence of the dogma of aggressive filling in the burn and the consequence of the automatic administration of a unit of crystalloid to each patient. This should question our practices in terms of volume expansion, especially for patients suffering small burns, from whom capillary leak should not be predominant but hypothermia could happen [31, 32]. However, the accuracy of very early fluid resuscitation remain, to our knowledge, a topic of debate. Studies already addressing this question in the adult [28] or paediatric [14] population found a trend towards more complications in situations of inadequate prehospital fluid resuscitation, but no impact on major side effects could not be definitely concluded.

Described as a critical topic and frequent challenge in the acute care of burned patients [33], pain management was reported in almost three-quarters of our caseload, which is quite similar to the 79% reported in another prehospital study [26]. The median pain on site was more intense for the large burns group (severe pain) as compared with the small burns group (moderate pain). Fentanyl was the most frequently used molecule. Ketamine has been used primarily in large burns (that had more severe pain) and most often (5/7) in combination with fentanyl. The doses of both fentanyl and ketamine correspond to the dosages recommended for acute analgesia or induction and maintenance of sedation [34–36]. Analgesics were used within the recommended doses, and the median pain score at emergency department arrival reached the minor pain category in both subgroups. However, variation stayed high and kept room for improvement. The low rate of initial pain assessment using the VNRS may partially be explained by altered consciousness, as 12 patients had a GCS < 14.

The helicopter PEMS was proportionally used more for large burns compared to small burns, as already shown in other similar prehospital caseload descriptions [37, 38]. The cause remains uncertain in our collective but is potentially linked to dispatch criteria and to the nature of the incidents themselves in peri-urban or rural areas. Longer prehospital intervention times were observed in patients with large burns, mainly due to an increase in on-site time, possibly caused by more extensive on-site support (more intravenous or intraosseous access and more intubations). Vital parameters of patients suffering large burns are slightly more pathological than those of patients suffering small burns, as shown elsewhere [26]. As expected, the evolution of these parameters during prehospital management tends to standardize [39], except the systolic blood pressure, which fell in both groups at the measurement at hospital arrival. A pressure drop during burn prehospital treatment has already been reported [27], and the role of opioid analgesic has been evoked [33]. Finally, more largely affected patients had higher NACA and ISS scores. They were more likely to be admitted to the ICU, and their ICU length of stay was longer, as was their hospital stay in general. These findings are similar to those of previous studies [26–28, 37].

### Limitations

This study is limited by its retrospective design, which may have influenced the quality of the data, as well as the presence of some missing data. The rigorous data collection process (standardized prehospital database and systematic review of each prehospital chart by a senior physician) may however counterbalance this limit.

Generalizability of our results may also be limited by the monocentric aspect of our study, which was carried out in a small mixed urban and peri-urban area with a high level of medical care and a prehospital system allowing paramedics a large degree of autonomy. However, this specific setting also has advantages relative to the uniformity and quality of the data, as it comes from an accredited burn centre, including notably standardized outcome adjudication. The relatively small median burned area in our collective constitutes in itself a limitation compared to other settings where dispatch criteria could influence the prevalence of more severe burns in greater numbers. Our small collective also limits the possibility of carrying out sub-group analyzes, for example on inhalation lesions or concomitant poisoning, which prompts further research. The few previous prehospital studies on these topics are indeed retrospective and include a relatively low number of patients. Therefore, our study brings additional scientific knowledge about this very specific and highly specialized type of patient management.

## Conclusion

The present study, which included 86 patients, provides further information about the management of burned patients in the prehospital setting. We found good agreement between the prehospital and hospital burn size estimations in most cases. As the differences between prehospital and hospital TBSA estimations were higher for large burns, these differences were unlikely to have influenced either patients’ orientation or outcome. Despite the fair burn size estimation, the quantity of crystalloid infused within prehospital care was higher than the recommended amount, suggesting a potential risk for fluid overload. It was probably of no consequence observed in our collective due to the brevity of the transport to the reference centre. Most patients benefited from systemic analgesia with either fentanyl or ketamine, but documentation of the pain intensity was lacking in about one-third of the patients. These results show that some progress can still be made in PEMS management of burned patients and emphasized the need for dedicated guidelines and modern decision aids to improve the systematic prehospital management of burns.

## Supplementary information

**Additional file 1: Figure S1.** Bland-Altman analysis of the differences between the hospital and prehospital TBSA estimations. The mean value between the two estimations is plotted against their difference. The mean difference between the hospital and prehospital TBSA estimations was − 0.154. The lower and upper limits of agreement were − 7.5 and 7.2%, respectively.

## Data Availability

The dataset analysed during the current study is available from the corresponding author on reasonable request.

## Abbreviations

CRT: Capillary refill time
GCS: Glasgow coma scale
ICU: Intensive care unit
IQR: InterQuartile range
ISS: Injury severity score
NACA score: National advisory committee for aeronautics score
PEMS: Physician-staffed emergency medical service
SpO_2_: Peripheral capillary oxygen saturation
TBSA: Total burn surface area
VNRS: Verbal numeric rating scale
