# Supplementary material for: Prehospital management of burns requiring specialized burn centre evaluation: a single physician-based emergency medical service experience
Source: Scand J Trauma Resusc Emerg Med. 2020 Aug 20;28:84. doi: 10.1186/s13049-020-00771-4 (PMC7439538; doi:10.1186/s13049-020-00771-4)
Supplement: Supplementary file 1 — Additional file 1: Figure S1. Bland-Altman analysis of the differences between the hospital and prehospital TBSA estimations. The mean value between the two estimations is plotted against their difference. The mean difference between the hospital and prehospital TBSA estimations was − 0.154. The lower and upper limits of agreement were − 7.5 and 7.2%, respectively. [file 13049_2020_771_MOESM1_ESM.docx]

Figure S1. Bland-Altman analysis of the differences between the hospital and prehospital TBSA estimations. The mean value between the two estimations is plotted against their difference. The mean difference between the hospital and prehospital TBSA estimations was -0.154. The lower and upper limits of agreement were –7.5% and 7.2%, respectively.
